# Self-Reported Visual Perceptual Abnormalities Are Strongly Associated with Core Clinical Features in Psychotic Disorders

**DOI:** 10.3389/fpsyt.2018.00069

**Published:** 2018-03-12

**Authors:** Brian P. Keane, Lisa N. Cruz, Danielle Paterno, Steven M. Silverstein

**Affiliations:** ^1^University Behavioral Health Care, Rutgers University, Piscataway, NJ, United States; ^2^Robert Wood Johnson Medical School, Rutgers University, Piscataway, NJ, United States; ^3^Center for Cognitive Science, Rutgers University, Piscataway, NJ, United States

**Keywords:** basic symptoms, visual perception, premorbid functioning, hallucinations, delusions, Bonn Scale, schizophrenia, depressive symptoms

## Abstract

**Background:**

Past studies using the Bonn Scale for the Assessment of Basic Symptoms (hereafter, Bonn Scale) have shown that self-reported perceptual/cognitive disturbances reveal which persons have or will soon develop schizophrenia. Here, we focused specifically on the clinical value of self-reported *visual* perceptual abnormalities (VPAs) since they are underexplored and have been associated with suicidal ideation, negative symptoms, and objective visual dysfunction.

**Method:**

Using the 17 Bonn Scale vision items, we cross-sectionally investigated lifetime occurrence of VPAs in 21 first-episode psychosis and 22 chronic schizophrenia/schizoaffective disorder (SZ/SA) patients. Relationships were probed between VPAs and illness duration, symptom severity, current functioning, premorbid functioning, diagnosis, and age of onset.

**Results:**

Increased VPAs were associated with: earlier age of onset; more delusions, hallucinations, bizarre behavior, and depressive symptoms; and worse premorbid social functioning, especially in the childhood and early adolescent phases. SZ/SA participants endorsed more VPAs as compared to those with schizophreniform or psychotic disorder-NOS, especially in the perception of color, bodies, faces, object movement, and double/reversed vision. The range of self-reported VPAs was strikingly similar between first-episode and chronic patients and did not depend on the type or amount of antipsychotic medication. As a comparative benchmark, lifetime occurrence of visual hallucinations did not depend on diagnosis and was linked only to poor premorbid social functioning.

**Conclusion:**

A brief 17-item interview derived from the Bonn Scale is strongly associated with core clinical features in schizophrenia. VPAs hold promise for clarifying diagnosis, predicting outcome, and guiding neurocognitive investigations.

## Introduction

“Basic symptoms”—as coined by Gerd Huber in 1989 (1)—are cognitive, perceptual, affective, proprioceptive, and social disturbances that are commonly though not exclusively reported in individuals with serious mental illness (2–4). Basic symptoms—also referred to as sub-clinical features or subjective cognitive dysfunction—are subtle experiential changes that are typically unobservable by others. They have been richly documented via in-depth clinical interviews with schizophrenia patients at least since the 1960s (5). Examples include thought interference, changes in object size or color, blurred vision, electrical sensations, and changes in emotional responses, among many others. A number of instruments have been used to measure basic symptoms but the Bonn Scale for the Assessment of Basic Symptoms (BSABS; hereafter the “Bonn Scale”) (2) remains arguably the most well-known.

Basic symptoms are worthwhile to investigate because they serve as a type of bellwether, preceding the attenuated psychotic symptoms, which themselves precede acute psychosis (3, 6–9). As an example, Klosterkotter and colleagues (10) found that—within 110 prodromal and 50 non-prodromal patients—70% of patients with at least one basic symptom developed schizophrenia whereas 96% of those without such symptoms did not. In a retrospective study on first-episode psychosis patients, people who converted to schizophrenia first experienced basic symptoms and attenuated psychotic symptoms, and then reported transient psychotic symptoms (11). In a prospective study with an 18-month follow-up, cognitive-perceptual basic symptoms and ultra-high-risk criteria jointly predicted first-episode psychosis (positive predictive value = 80%; AOC = 81%) (12).

Basic symptoms are also worthwhile to investigate because they may occur more often in schizophrenia than other related disorders, potentially providing valuable diagnostic information when diagnosis is unclear (5, 13–15). For example, according to Ricca et al. (15), a schizophrenia group showed the highest total basic symptom scores, a bipolar group reported the lowest scores, and the schizoaffective group’s scores fell in between, so that the only significant difference was between the schizophrenia and bipolar groups. In a cross-sectional study, Meng et al. (3) found that the rate of endorsing at least one basic symptom was lowest for a general adolescent population (30.4%), intermediate for a non-psychotic first-episode group (81%), and highest for a psychotic first-episode group (97%).

In the present investigation, we focus on *visual* basic symptoms because visual distortions are associated with increased suicidal ideation in help-seeking adolescents (16), increased negative symptoms in remitted outpatients (17), and increased psychosis risk among prodromal patients (10). Moreover, the visual system and its underlying neurobiology is arguably the best understood in the human brain. Visual disturbances, once identified, can help reveal the pathways and circuits that are most affected by the illness (18). Although visual perceptual abnormalities (VPAs) occur in over 60% of patients (19), they have been studied far less often than other symptoms such as formal thought disorder or cognitive disturbances, even though there is ample laboratory evidence of visual processing impairments in schizophrenia patients and high-risk samples (18, 20–22). Therefore, our goal was to clarify the clinical significance of VPAs in schizophrenia and schizophrenia spectrum disorders.

In the present study, we asked patients with first-episode psychosis or chronic schizophrenia/schizoaffective disorder whether 17 VPAs had ever been experienced. One goal was simply to consider whether VPAs would become more diverse with illness duration. An affirmative answer was expected partly because older, chronic patients have more opportunity to experience such symptoms and also because certain visual deficits worsen with illness chronicity, including contrast sensitivity, surround suppression, and contour integration (23–25). A second consideration was whether Bonn Scale scores differed between patients with schizophrenia or schizoaffective disorder (SZ/SA) versus those with other psychotic disorders (“OtherPsy”). Schizophrenia and schizoaffective disorder patients were treated jointly given the tenuous clinical distinction between the two (26) and given that the two diagnostic groups have been shown to express similar basic symptom levels (15). On the basis of previous research, an affirmative answer was again expected (3, 10, 13). Lastly, in an exploratory analysis, we investigated whether VPAs were related to age of onset, premorbid adjustment, current functioning, and recent symptoms.

## Materials and Methods

### Participants

The study sample consisted of 22 chronic patients with schizophrenia (*N* = 16) or schizoaffective disorder (*N* = 6); and 21 first-episode psychosis patients with schizophrenia (*N* = 5), schizoaffective disorder (*N* = 4), schizophreniform disorder (*N* = 5), and psychotic disorder not otherwise specified (*N* = 7) (Tables 1 and 2). For all subjects, inclusion/exclusion criteria were (1) age 18–65; (2) no electroconvulsive therapy in the past 8 weeks; (3) no neurological or pervasive developmental disorders; (4) no substance dependence in the last 6 months as assessed with the Mini International Neuropsychiatric Interview 6.0 (MINI) (27); (5) no brain injury due to accident or illness (e.g., stroke or brain tumor); (6) no amblyopia (as assessed by informal observation and self-report); (7) visual acuity of 20/32 or better (with corrective lenses if necessary); and (8) sufficient spoken English to complete testing. The additional criteria for chronic patients were: having experienced at least two prior psychiatric hospitalizations and having received a DSM IV-TR diagnosis of SZ/SA at the time of testing. The additional criteria for the first-episode sample were: having had exactly one psychiatric hospitalization and having recently received a psychotic disorder diagnosis, in most cases within 1 year of testing. The first-episode status was confirmed by a review of medical records and a structured psychiatric interview, as described immediately below. All patients except one were on antipsychotic medication at the time of testing.

**Table 1:** Demographic and clinical characteristics of chronic and first-episode patients.

	Chronic (*N* = 22)		First episode (*N* = 21)		Group comparison
Variable	Mean or percent	SD	Mean or percent	SD	*p* (uncorrected)
Age (years)	38.1	13.6	25.6	6.6	*p* < 0.001
Education, parental average (years)	13.2	4.7	12.97	2.7	0.85
Education, self (years)	13.6	4.5	13.6	2.01	0.99
FSIQ (Shipley)	90.1	13.4	92.5	17.3	0.62
Gender (% male)	63.6%		71.4%		0.32
Handedness (% right)	81.8%		100.0%		0.04
Antipsychotic type: typical/atypical/both (%)	35/45/15		33/61/6		0.649
Chlorpromazine equiv. (mg/day)	498.0	361.6	352.4	237.0	0.15
Functioning, current (MSIF)	4.19	0.9	4.5	1.0	0.33
Functioning, premorbid (PAS)	0.4	0.1	0.3	0.1	0.50
Illness duration (years)	18.9	12.4	4.0	8.0	*p* < 0.001
Illness onset age (years)	19.3	6.9	21.9	5.3	0.17
Bonn Vision Total (VPAs)	3.0	3.4	2.2	4.0	0.49
PANSS, 5-Factor Positive	9.6	5.2	7.9	4.4	0.28
PANSS, 5-Factor Negative	12.2	3.7	14.1	4.8	0.16
PANSS, 5-Factor Disorganized	7.6	2.8	6.5	2.7	0.22
PANSS, 5-Factor Excited	5.9	2.4	5.6	2.5	0.73
PANSS, 5-Factor Depressed	8.6	3.4	7.0	4.0	0.45
PANSS Total	60.5	17.6	58.1	13.3	0.63
SAPS Hallucinations	6.9	8.3	4.0	5.7	0.20
SAPS Delusions	11.1	7.6	6.0	8.8	0.05
SAPS Bizarre Behavior	2.8	3.0	2.0	2.8	0.38
SAPS Formal Thought Disorder	5.4	7.6	3.1	5.8	0.28

*FSIQ, Full-Scale IQ; MSIF, Multidimensional Scale of Independent Functioning; PAS, Premorbid Adjustment Scale; PANSS, Positive and Negative Syndrome Scale; SAPS, Scale for the Assessment of Positive Symptoms*.

*The MSIF score reflects the global level of current functioning; the PAS score reflect premorbid functioning averaged across age periods*.

**Table 2:** Demographic and clinical characteristics of SZ/SA and OtherPsy participants.

	SZ/SA (*N* = 31)		OtherPsy (*N* = 12)		Group comparison
Variable	Mean or percent	SD	Mean or percent	SD	*p* (uncorrected)
Age (years)	35.5	12.8	23.1	3.9	*p* < 0.001
Education, parental average (years)	13.1	4.2	13.2	2.7	0.96
Education, self (years)	13.6	4.0	13.5	1.7	0.93
FSIQ (Shipley)	89.2	15.2	96.8	14.8	0.16
Gender (% male)	70%		70%		0.56
Handedness (% right)	90%		100%		0.20
Antipsychotic type: typical/atypical/both (%)	32/50/14		40/60/0		0.32
Chlorpromazine equiv. (mg/day)	459.8	325.3	343.0	275.1	0.75
Functioning, current (MSIF)	4.3	0.9	4.5	0.9	0.46
Functioning, premorbid (PAS)	0.3	0.1	0.3	0.1	0.31
Illness duration (years)	15.5	12.9	0.9	1.6	*p* < 0.001
Illness onset age (years)	19.9	6.8	22.4	3.9	0.24
Bonn Vision Total (VPAs)	3.2	4.1	0.8	1.3	0.006
PANSS, 5-Factor Positive	9.6	5.0	6.5	3.7	0.07
PANSS, 5-Factor Negative	12.6	4.2	14.5	4.3	0.20
PANSS, 5-Factor Disorganized	7.3	2.9	6.2	2.3	0.27
PANSS, 5-Factor Excited	6.0	2.5	5.3	2.4	0.43
PANSS, 5-Factor Depressed	8.1	3.6	7.1	4.1	0.45
PANSS Total	60.6	16.7	55.9	11.8	0.40
SAPS Hallucinations	6.9	7.8	1.5	2.8	0.003
SAPS Delusions	10.4	8.5	3.6	6.5	0.02
SAPS Bizarre Behavior	2.9	3.1	1.1	1.7	0.02
SAPS Formal Thought Disorder	4.6	7.1	3.3	6.3	0.59

*The antipsychotic type variable excludes one subject not on medication*.

*FSIQ, Full-Scale IQ; MSIF, Multidimensional Scale of Independent Functioning; PAS, Premorbid Adjustment Scale; PANSS, Positive and Negative Syndrome Scale; SAPS, Scale for the Assessment of Positive Symptoms*.

*The MSIF score reflects the global level of current functioning; the PAS score reflect premorbid functioning averaged across age periods*.

### Procedure

Psychiatric diagnosis was assessed with the Structured Clinical Interview for DSM-IV (28) and supplemented with electronic medical records when the diagnosis was unclear. All clinical instruments (including the Bonn Scale) were administered by an experienced rater (DP), who had established reliability with raters in other ongoing studies (ICC > 0.8). Intellectual functioning of all subjects was assessed with a brief vocabulary test that correlates highly (*r*~0.8) with WAIS-III verbal IQ scores (29) and that provides an estimate of premorbid IQ in patients.

The full semi-structured Bonn Scale interview consists of 169 items and six subsections (2); the Scale has been validated using a sample of 243 patients and 79 healthy individuals (30). Because our aim was to probe for anomalous *visual* experiences, we administered the vision subsection only, which probes lifetime history of 17 specific VPAs (for a complete list, please see Table S1 in Supplementary Material). Prior studies have shown that the visual subsection generates good inter-rater reliability (31). A symptom was not counted as being present if it could be explained by drug use, a general medical condition, or hypnopompic or hypnogogic states. Participants were given a score of “1” or “0” if a visual abnormality was or was not present, respectively, and “0.5” if the symptom was questionable.

The Scale for the Assessment of Positive Symptoms (SAPS) (32) assessed positive symptoms within the past 2 weeks, and considered four symptom domains—hallucinations, delusions, bizarre behavior, and positive formal thought disorder. Scores from each domain reflect the summed score for the questions within that domain. The Positive and Negative Syndrome Scale (PANSS) (33) provided information about symptom severity over the last two weeks. We report PANSS scores from a “consensus” five-factor model (Positive, Negative, Disorganized/Concrete, Depressed, Excited),^1^ which was constructed from 29 previous five-factor models, validated with a 600 patient sample, further confirmed with an international sample of ~2,500 patients, and shown to be superior to the three-factor model in confirmatory factor analyses (34, 35). The Premorbid Adjustment Scale (PAS) (36) measured sociability, peer relationship quality, scholastic performance, school adaptation, and (where appropriate) social-sexual functioning up to 1 year before illness onset; this was done for childhood (ages 6–11), early adolescence (ages 12–15), late adolescence (ages 16–18), and adulthood (ages 19 and above). “Illness onset” on this scale refers to when a patient first noticed and became troubled by one or more positive symptoms (37); illness duration was defined as the time elapsed between illness onset and clinical assessment. Because illness onset is defined as when the first positive symptom emerges rather than when the person first meets criteria for a psychotic disorder, a few subjects ended up having onset ages in early childhood. To keep faithful to our original data collection method and to yield a maximally inclusive sample, we continued to include these subjects in the final analysis. In line with what others have done, the PAS General score was not included since it is reflective of functioning before and after illness onset (37); see also (38, 39). The Multidimensional Scale of Independent Functioning (MSIF) independently evaluated role position, support, and performance in work, education, and home life (in decreasing order of emphasis) within the month before the interview (40).

Not all subjects provided data for all measures. Some subjects were either unable to unwilling to answer particular questions, or complete a study session. For the SAPS and PANSS, there were 41 cases, except for the Excited and Disorganized PANSS factors, which had 39 and 40 cases, respectively; for the MSIF, there were 43 cases; and for the PAS, there were 39 cases. The PAS provided the ages of onset for all 39 subjects and the medical records provided such information for two additional patients (for a total N of 41). Three of the PAS subjects had an age of onset before the designated childhood period (<age 7) and, thus, did not yield any data on premorbid functioning. Each type of functioning (e.g., scholastic performance) derived from 36 subjects, except the social/sexual category, which consisted of 35 subjects. Later age ranges necessarily had fewer subjects with premorbid functioning data, and so the number of data points for childhood, early adolescence, late adolescence, and adulthood was 36, 36, 32, and 21, respectively.

### Data Analyses

Visual perceptual abnormalities were operationalized as the total score on the visual subsection of the Bonn Scale, with higher scores denoting more severe disturbances. *T*-tests were two-tailed and equal variances were assumed, unless a significant Levene’s test dictated otherwise. Relationships between clinical measures and VPAs were evaluated with a series of simple linear regression analyses, using the VPA count totals as the independent variable. (Note that because VPAs serve as an explanatory rather than a response variable, issues with possible zero-inflation or over-dispersion are not further considered.) To reduce Type I errors, a False Discovery Rate controlling procedure (*q* < 0.05) was used for the exploratory regression analyses (41), which included the five PANSS factors, the four SAPS domain scores, the four current functioning scores (including a “global” score; MSIF), and the 10 premorbid functioning (PAS) scores (including age of onset). An FDR correction was also applied to the 18 *t*-tests on the Bonn Scale items, including the overall score.

## Results

**Figure 1:**
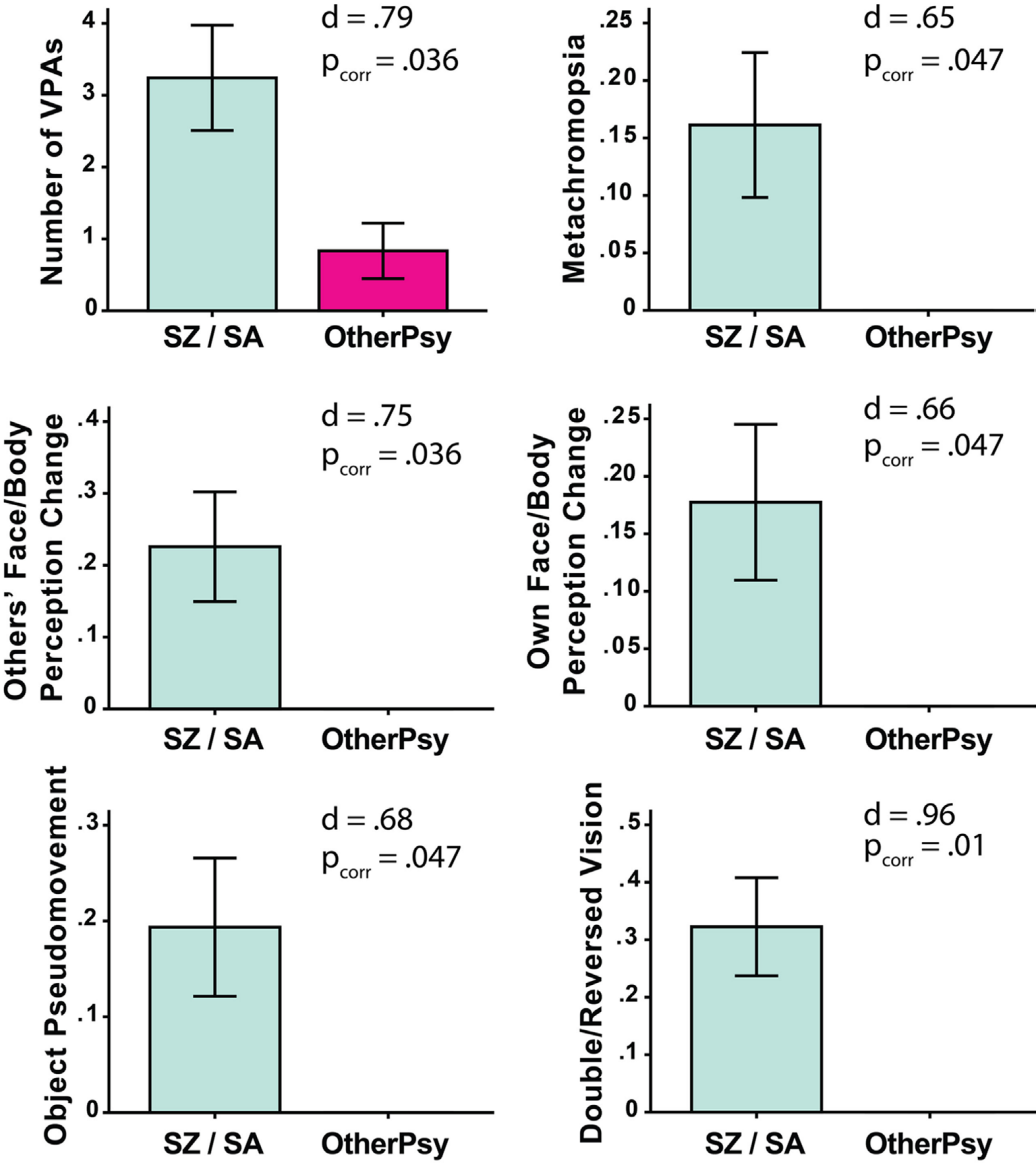
Significant between-group differences on total number of self-reported visual perceptual abnormalities (VPAs) as measured with the Bonn Scale. The schizophrenia/schizoaffective (SZ/SA) group endorsed more VPAs than the group with other psychotic disorders (OtherPsy). The *p*-values are FDR corrected. Error bars denote ± SEM.

Of the 43 patients, 27 (63%) reported at least one VPA.^2^ The total number of VPAs did not differ between the chronic and first episode groups [Chronic: M = 2.95, SD = 3.45; First Episode: M = 2.17, SD = 3.95; *t*(39.6) = 0.695, *p* = 0.49]. At the same time, the SZ/SA group endorsed significantly more VPAs as compared to the OtherPsy group [SZ/SA: M = 3.24, SD = 4.08; OtherPsy: M = 0.83, SD = 1.34; *t*(40.47) = 2.91; *p_corr_* = 0.036; *d* = 0.79] (Figure 1). The specific VPAs that distinguished the groups were: metachromopsia [*t*(30) = 2.56; *p*_corr_ = 0.047; *d* = 0.65], change in face/body perception [*t*(30) = 2.96; *p*_corr_ = 0.036; *d* = 0.75], changes in own face perception [*t*(30) = 2.62; *p*_corr_ = 0.047; *d* = 0.66], object pseudomovement [*t*(30.0) = 2.68; *p*_corr_ = 0.047; *d* = 0.68], and double/reversed vision [*t*(30.0) = 3.78; *p*_corr_ = 0.01; *d* = 0.96]. Although age and illness duration differed between groups, these factors were not associated with the total or individual Bonn Scale scores.

**Figure 2:**
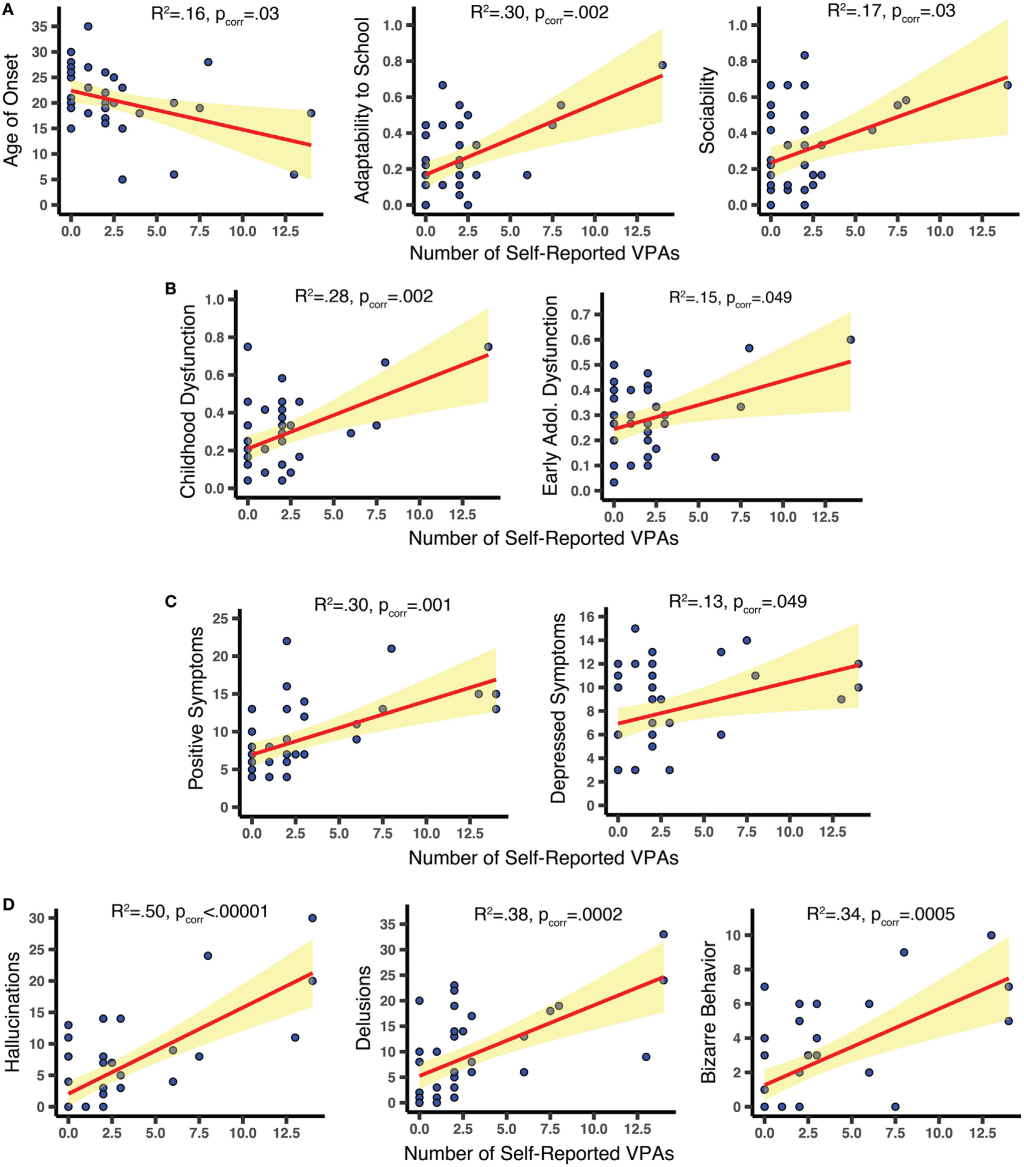
Scatterplots relating clinical features to number of self-reported visual perceptual abnormalities (VPAs). **(A,B)** Using the Premorbid Adjustment Scale, we found that increased VPAs were related to earlier age of onset of psychotic symptoms and worse premorbid sociability, adaptability to school, childhood functioning, and early adolescent functioning. **(C)** Increased VPAs were associated with more severe positive and depressed Positive and Negative Syndrome Scale symptoms within 2 weeks of assessment. **(D)** Results from the SAPS demonstrate that increased VPAs were linked to increased hallucinations, delusions, and bizarre behavior within 2 weeks of assessment. All effects were derived from FDR-corrected simple linear regression analyses. Confidence bands show the 95% confidence intervals for the fitted regression lines.

Results from the simple linear regression analyses are shown in Figure 2. Among the 10 PAS values considered, increased visual abnormalities were associated with more premorbid dysfunction (higher PAS scores) for Sociability [*R*^2^ = 0.17; *F*(1,34) = 7.05, *p*_corr_ = 0.03] and Adaptability to school [*R*^2^ = 0.30, *F*(1,34) = 14.3, *p*_corr_ = 0.002]; effects were strongest for Childhood [*R*^2^ = 0.28, *F*(1,34) = 13.11, *p*_corr_ = 0.003] and Early Adolescence [*R*^2^ = 0.15, *F*(1,34) = 6.06, *p*_corr_ = 0.049]. Subjects who reported more VPAs also reported earlier onset of psychotic symptoms [*R*^2^ = 0.163, *F*(1,39) = 7.58, *p*_corr_ = 0.03]. VPAs were not significantly associated with current levels of functioning (all *p*_corr_ > 0.22). On the PANSS, more VPAs predicted more positive symptoms [*R*^2^ = 0.30, *F*(1,39) = 16.78, *p*_corr_ = 0.001] and depressed symptoms [*R*^2^ = 0.13, *F*(1,39) = 5.74, *p*_corr_ = 0.049], but had little relation to negative, disorganized, or excited symptoms (*p*_corr_ > 0.39). On the SAPS, we found correlations with: hallucinations [*R*^2^ = 0.50, *F*(1,39) = 38.88, *p*_corr_ < 0.00001], delusions [*R*^2^ = 0.38, *F*(1,39) = 23.86; *p*_corr_ = 0.0002], and bizarre behavior [*R*^2^ = 0.34, *F*(1,39) = 20.05, *p*_corr_ = 0.0005] but not with positive formal thought disorder (*p_cor_*_r_ > 0.39). Even when the last two SAPS Hallucination items were removed—so that only non-visual hallucinations were assessed—the SAPS Hallucination dimension was still significantly associated with VPAs [*R*^2^ = 0.44, *F*(1,38) = 30.08; *p* < 0.00001]. Note that heteroscedasticity was not significantly impacting any of the 10 significant effects (Breusch Pagan Test; all *p* > 0.06, before statistical correction).

Antipsychotic medication effects cannot readily explain the results. The Bonn Scale scores did not depend on antipsychotic medication type (none, atypical, typical) [*F*(2,34) = 0.32; *p* = 0.73] and were not associated with chlorpromazine equivalent doses [*r*(36) = 0.18; *p* = 0.23]. Despite differing on VPAs, the SZ/SA group and the OtherPsy group did not differ on antipsychotic type (*p* = 0.75, *Fisher’s Exact Test*) or chlorpromazine equivalent values [*t*(36) = 1.01; *p* = 0.32]. When a CPZ equivalent variable was added to VPAs as a predictor within a stepwise AIC regression, the latter was always included in the final model while the CPZ variable was not (one dubious exception was that the CPZ equivalent values were also not included in the model of the PAS Childhood score).

The scatterplots in Figure 2 indicate that the subjects with the most extreme Bonn Scale scores could be driving the effects. To explore the possibility, we removed individuals (*N* = 3) whose leverage values were 2.5 times the average and re-ran the analyses. The PANSS Depressed symptoms, the SAPS Bizarre symptoms, and the PAS Adaptation to School and Childhood functioning scores were all significant before statistical correction, but not after (*p*s < 0.03, *p*s_corr_ > 0.10). Surviving statistical correction were relationships with the PANSS Positive [*R*^2^ = 0.29, *F*(1,36) = 14.88; *p*_corr_ = 0.007] and the SAPS Hallucinations and Delusions scores [*R*^2^ = 0.27, *F*(1,36) = 13.15, *p*_corr_ = 0.006; *R*^2^ = 0.45, *F*(1,36) = 12.87, *p*_corr_ = 0.006]. Note that the high-leverage data points did not overly influence the fitted regression lines (Cook’s distance values < 1.0) and were generally not outliers (studentized residuals < 2.5 SD).^3^ Therefore, we think it is reasonable to include these subjects into the final analysis. Nevertheless, these data suggest that the most perceptually disturbed participants drove some, but certainly not all, of the effects.

Does the Bonn Scale visual score tells us anything over and above what we could learn from lifetime occurrence of visual hallucinations? To consider the question, we examined 35 subjects with available SCID visual hallucination data, and found that 48.6% endorsed visual hallucinations, 48.6% endorsed no such hallucinations, and one subject was indeterminate. Our somewhat higher rate of visual hallucinations falls within the bounds of what has been reported in earlier studies [see Table 1 of Ref. (42)]. Re-running the same analyses as before, we found that the SZ/SA and OtherPsy groups reported similar number of lifetime visual hallucinations [*t*(33) = 1.13; *p* = 0.27; *d* = 0.43]. For the regression analyses, we found that only the PAS sociability [*R*^2^ = 0.28; *F*(1,27) = 10.40, *p*_corr_ = 0.04] and PAS adulthood [*R*^2^ = 0.44; *F*(1,17) = 13.53, *p*_corr_ = 0.04] were related to lifetime visual hallucinations; all other variables bore no association (all *R*^2^ < *0.19*; all *p*_corr_ > 0.09). Switching to one-tail tests to improve sensitivity did not qualitatively alter the foregoing results, with the one exception being that SAPS Hallucination score became significant (*R*^2^ = 0.186, *p*_corr_ = 0.047). The overall smaller effects could be because visual hallucinations were measured from only a single SCID question, reducing its reliability as a construct; or it could be because visual hallucination endorsements are stigmatized and harder to accurately obtain during interviews. Either way, VPAs provide insights over and above from what can be accumulated from simply examining lifetime visual hallucinations.

## Discussion

We investigated whether VPAs as assessed with the Bonn Scale were related to core clinical features in patients with psychosis. It was found that illness duration had little bearing on VPAs, that schizophrenia/schizoaffective disorder patients reported more VPAs than those with other psychotic disorders, and that VPAs were associated with worse premorbid functioning, earlier age of onset, more depressive symptoms, and more positive symptoms (PANSS). The effects were often large in magnitude, could not be explained in terms of antipsychotic medication effects, and could generally not be duplicated if visual hallucinations rather than VPAs were inserted into the analyses.

### Implications

Certain implications are worth considering more closely. First, the SZ/SA group endorsed more VPAs than the OtherPsy group even though the groups were undifferentiated on PANSS symptoms and current functioning levels. The finding fits with longitudinal and cross-sectional investigations on visual and non-visual basic symptoms (3, 10, 13, 15), and underscores the utility of VPAs for clarifying current diagnosis and predicting outcomes. Somewhat surprisingly, illness duration had no relation to VPAs. This could be because chronic patients are largely stabilized and treated following their first psychotic break or it could be because such symptoms generally no longer morph in character once the illness first emerges. Given that VPAs were associated with poorer childhood adjustment and earlier age of onset, a likely possibility is that VPAs act as an early warning sign, arising first in the earliest prodromal stages and subsiding significantly after psychosis onset. Future studies will have to trace the developmental time course and causal interrelationship between VPAs and clinical variables, from the prodromal phase onward.

The visual system is arguably the most well understood in the human brain and so self-reported VPA reports can yield important clues as to what circuits or mechanisms are differentially impacted by the disorder. Very few studies have directly linked VPAs to psychophysical results, but Kéri and colleagues (17) found that increased anomalous experiences correlated with worse early visual contour linking, worse rapid scene categorization, worse detection of the offset direction of alternatively flickering sinusoidal gratings (1 c/d), and worse discrimination of the relative alignment of two low contrast dots. The latter three were thought to stem from dysfunction along the magnocellular pathway but the assertion remains contentious (43). Our findings of more abnormal perception of bodies, faces, and object motion in psychosis dovetail with earlier findings of abnormal visual behavioral performance and abnormal brain activation in associated brain structures, namely medial temporal cortex (motion), fusiform gyrus (faces), and posterior superior temporal sulcus (bodies) (44–48). Disturbed color perception has been documented in earlier self-report studies (19, 49, 50); double or reversed vision has been less well supported. Psychophysical and neural evidence for either disturbance is generally weak and merit further investigation.

Our results seemingly clash with Kéri and colleagues (17), who found that VPAs positively correlated with negative but not positive symptoms. Why the discrepancy? We first note that although the PANSS Negative five-factor score did not correlate with VPAs, the PANSS “Depressed” score *did* correlate [*r*(39) = 0.32; *p*_corr_ < 0.04], indicating that only certain types of negative symptoms may be driving the effect. Moreover, the patients of Keri et al. had, as a whole, extremely minimal positive symptoms [mean total SAPS = 8.8 (3.8 SD)] whereas our sample had moderate levels of such symptoms [mean total SAPS = 20.7 (18.7 SD)]. Increased positive symptom heterogeneity is probably necessary to detect any relation with VPAs.

The Bonn vision subscale has noteworthy methodological advantages. It probes seemingly benign visual changes, implying that patients can potentially be less guarded in fully and candidly acknowledging the queried symptoms. The subscale also taps into lifetime VPA occurrence with a binary response format, freeing patients from having to remember the graded frequency, duration, time frame, or life impact of the symptoms, all of which can be clouded by memory impairment or experimenter demand bias (51). In addition, the subscale is straightforward to administer: it does not require the clinician to make fine-grained symptom evaluations and takes only about 20 min to complete.

### Limitations and Concluding Remark

A limitation is that the OtherPsy group had a small number of subjects (*N* = 12), making it unclear whether our results can generalize to a larger and more variegated sample of psychotic individuals without schizophrenia/schizoaffective disorder. Although the 10 significant regression effects could survive FDR correction and although they were generally of large magnitude and of a direction that could be anticipated by past literature, it would still be useful to confirm the findings with a larger patient sample. In addition, anticholinergic medication effects on visual perception are possible (52) but not often considered in schizophrenia research; it would, therefore, be worthwhile to probe for relationships between anticholinergic load and VPA susceptibility (53). We also did not screen subjects for lifetime hallucinogen use, which in rare cases can lead to distressing and persisting visual distortions months or even years after the first intoxication—Hallucinogen Persisting Perception Disorder (HPPD). This concern is mitigated by a few facts: (i) subjects were excluded if they had a substance dependence within 6 months of participation; (ii) no subjects were given a diagnosis of substance-induced psychotic disorder; (iii) VPAs were not counted if they could be attributed to drug exposure; and (iv) according to initial estimates, only 4% of individuals who try hallucinogens ultimately receive a diagnosis of HPPD (see DSM-5, p. 531). Nevertheless, a more conservative approach going forward would be to exclude individuals with any history of hallucinogen use. Another limitation is that although there is good reason to focus on *visual* disturbances, other sense modalities may prove to be similarly valuable and should be more closely inspected in future investigations. Finally, incorporating a control group could have provided a baseline against which to compare patients.

Limitations aside, the current investigation demonstrates that self-reported VPAs unequivocally flag earlier onset age, more positive symptoms, more depressive symptoms, and poorer premorbid functioning. VPAs were more prominent in schizophrenia/schizoaffective disorder as compared to other psychotic disorders and do not continue to proliferate in character following the first psychotic break. The effects, when significant, were often large in magnitude (Cohen’s *ds* > 0.9; *rs* > 0.5) and could not be attributed to antipsychotic medication effects. Thus something as basic as abnormal visual experiences—surveyed with 17 simple yes/no questions—yields surprising insight into clinically important illness features. Future investigations will need to elaborate on the neural and psychophysical mechanisms underlying these changes as well as their promise for predicting conversion, clarifying diagnosis, and improving treatment.

## Ethics Statement

The Rutgers University Institutional Review Board approved the study, and written informed consent was obtained from all subjects. All procedures contributing to this work comply with the ethical standards of the relevant national and institutional committees on human experimentation and with the Helsinki Declaration of 1975, as revised in 2008. All participants received monetary compensation for their time.

## Author Contributions

BK and SS designed the study and wrote the protocol. DP and BK collected the data. BK and LC analyzed the data. LC and BK prepared the figures and tables and conducted the literature searches. LC, BK, SS, and DP wrote-up the paper. BK and SS responded to the reviewers. All authors contributed to and have approved the final manuscript.

## Conflict of Interest Statement

The authors declare that the research was conducted in the absence of any commercial or financial relationships that could be construed as a potential conflict of interest.

## Footnotes

^1^For clarity, the Disorganized/Concrete factor will simply be referred to as “Disorganized”.

^2^Subjects with and without VPAs were largely the same, having similar ages, education levels (self + parental average), IQ, and gender ratio, and receiving similar levels of antipsychotic dose (CPZ equivalents) (all *p*s > 0.10 before correction for multiple comparisons).

^3^One exception is that there was a single high-leverage outlier for the SAPS Delusion analysis (panel D, middle graph); however, removing this data point would strengthen the effect.

## References

[1] Huber G Gross G. The concept of basic symptoms in schizophrenic and schizoaffective psychoses. Recenti Prog Med (1989) 80(12):646–52. 2697899

[2] Gross G Huber G Klosterkotter J Linz M Bonn Scale for the Assessment of Basic Symptoms (BSABS). Berlin: Springer (1987).

[3] Meng H Schimmelmann BG Koch E Bailey B Parzer P Günter M Basic symptoms in the general population and in psychotic and non-psychotic psychiatric adolescents. Schizophr Res (2009) 111:32–8. 10.1016/j.schres.2009.03.001 19321309

[4] Gross G Huber G The history of the basic symptom concept. Acta Clin Croat (2010) 49(Suppl):47–59.

[5] Schultze-Lutter F. Subjective symptoms of schizophrenia in research and the clinic: the basic symptom concept. Schizophr Bull (2009) 35:5–8. 10.1093/schbul/sbn139 19074497 PMC2643966

[6] Fusar-Poli P Borgwardt S Bechdolf A Addington J Riecher-Rössler A Schultze-Lutter F The psychosis high-risk state. JAMA Psychiatry (2013) 70:107–14. 10.1001/jamapsychiatry.2013.269 23165428 PMC4356506

[7] Ruhrmann S Schultze-Lutter F Klosterkotter J. Early detection and intervention in the initial prodromal phase of schizophrenia. Pharmacopsychiatry (2003) 36:S162–7. 10.1055/s-2003-45125 14677074

[8] Schultze-Lutter F Ruhrmann S Fusar-Poli P Bechdolf A Schimmelmann BG Klosterkötter J. Basic symptoms and the prediction of first-episode psychosis. Curr Pharm Des (2012) 18:351–7. 10.2174/138161212799316064 22239566

[9] Huber G Gross G Schüttler R Linz M. Longitudinal studies of schizophrenic patients. Schizophr Bull (1980) 6:592–605. 10.1093/schbul/6.4.592 7444391

[10] Klosterkötter J Hellmich M Steinmeyer EM Schultze-Lutter F. Diagnosing schizophrenia in the initial prodromal phase. Arch Gen Psychiatry (2001) 58:158–64. 10.1001/archpsyc.58.2.158 11177117

[11] Schultze-Lutter F Ruhrmann S Berning J Maier W Klosterkotter J. Basic symptoms and ultrahigh risk criteria: symptom development in the initial prodromal state. Schizophr Bull (2010) 36:182–91. 10.1093/schbul/sbn072 18579555 PMC2800137

[12] Ruhrmann S Schultze-Lutter F Salokangas RKR Heinimaa M Linszen D Dingemans P Prediction of psychosis in adolescents and young adults at high risk: results from the prospective European prediction of psychosis study. Arch Gen Psychiatry (2010) 67:241–51. 10.1001/archgenpsychiatry.2009.206 20194824

[13] Parnas J Handest P Saebye D Jansson L. Anomalies of subjective experience in schizophrenia and psychotic bipolar illness. Acta Psychiatr Scand (2003) 108:126–33. 10.1034/j.1600-0447.2003.00105.x 12823169

[14] Raballo A Saebye D Parnas J. Looking at the schizophrenia spectrum through the prism of self-disorders: an empirical study. Schizophr Bull (2011) 37:344–51. 10.1093/schbul/sbp056 19528205 PMC3044618

[15] Ricca V Galassi F La Malfa G Mannucci E Barciulli E Cabras PL. Assessment of basic symptoms in schizophrenia, schizoaffective and bipolar disorders. Psychopathology (1997) 30:53–8. 10.1159/000285029 9042683

[16] Granö N Salmijärvi L Karjalainen M Kallionpää S Roine M Taylor P. Early signs of worry: psychosis risk symptom visual distortions are independently associated with suicidal ideation. Psychiatry Res (2015) 225:263–7. 10.1016/j.psychres.2014.12.031 25595340

[17] Kéri S Kiss I Kelemen O Benedek G Janka Z. Anomalous visual experiences, negative symptoms, perceptual organization and the magnocellular pathway in schizophrenia: a shared construct? Psychol Med (2005) 35:1445–1411. 10.1017/S0033291705005398 16164768

[18] Silverstein SM Keane BP. Perceptual organization impairment in schizophrenia and associated brain mechanisms: review of research from 2005 to 2010. Schizophr Bull (2011) 37:690–9. 10.1093/schbul/sbr052 21700589 PMC3122298

[19] Phillipson OT Harris JP. Perceptual changes in schizophrenia: a questionnaire survey. Psychol Med (1985) 15:859–66. 10.1017/S0033291700005092 4080889

[20] Silverstein SM Visual perception disturbances in schizophrenia: a unified model. Nebr Symp Motiv (2016) 63:77–132. 10.1007/978-3-319-30596-7_4 27627825

[21] Silverstein SM Rosen R Schizophrenia and the eye. Schizophr Res (2015) 2:46–55. 10.1016/j.scog.2015.03.004 PMC4559409 26345525

[22] Silverstein SM Keane BP. Vision science and schizophrenia research: toward a re-view of the disorder editors’ introduction to special section. Schizophr Bull (2011) 37:681–9. 10.1093/schbul/sbr053 21700588 PMC3122283

[23] Keane BP Paterno D Kastner S Silverstein SM. Visual integration dysfunction in schizophrenia arises by the first psychotic episode and worsens with illness duration. J Abnorm Psychol (2016) 125:543–9. 10.1037/abn0000157 27030995 PMC4850085

[24] Silverstein SM Keane BP Wang Y Mikkilineni D Paterno D Papathomas TV Effects of short-term inpatient treatment on sensitivity to a size contrast illusion in first-episode psychosis and multiple-episode schizophrenia. Front Psychol (2013) 4:466. 10.3389/fpsyg.2013.00466 23898311 PMC3721030

[25] Kiss I Fábián Á Benedek G Kéri S. When doors of perception open: visual contrast sensitivity in never-medicated, first-episode schizophrenia. J Abnormal Psychol (2010) 119:586–93. 10.1037/a0019610 20677847

[26] Cheniaux E Landeira-Fernandez J Lessa Telles L Lessa JLM Dias A Duncan T Does schizoaffective disorder really exist? A systematic review of the studies that compared schizoaffective disorder with schizophrenia or mood disorders. J Affect Disord (2008) 106:209–17. 10.1016/j.jad.2007.07.009 17719092

[27] Sheehan DV Lecrubier Y Sheehan KH Amorim P Janavs J Weiller E The Mini-International Neuropsychiatric Interview (MINI): the development and validation of a structured diagnostic psychiatric interview for DSM-IV and ICD-10. J Clin Psychiatry (1998). 9881538

[28] First M Spitzer R Gibbon M Williams J Structured Clinical Interview for DSM-IV-TR Axis I Disorders, Research Version, Patient Edition (SCID-I/P). New York: New York State Psychiatric Institute (1995).

[29] Wechsler D WAIS-III Wechsler Adult Intelligence Scale. San Antonio: Psychological Corporation (1997).

[30] Klosterkötter J Ebel H Schultze-Lutter F Steinmeyer EM Diagnostic validity of basic symptoms. Eur Arch Psychiatry Clin Neurosc (1996) 246:147–54. 10.1007/BF02189116 8739400

[31] Vollmer-Larsen A Handest P Parnas J. Reliability of measuring anomalous experience: the Bonn Scale for the Assessment of Basic Symptoms. Psychopathology (2007) 40:345–8. 10.1159/000106311 17657133

[32] Andreasen N Scale for the Assessment of Positive Symptoms (SAPS). Iowa City: University of Iowa (1984).

[33] Kay SR Fiszbein A Opler LA. The Positive and Negative Syndrome Scale (PANSS) for schizophrenia. Schizophr Bull (1987) 13:261–76. 10.1093/schbul/13.2.261 3616518

[34] Langeveld J Andreassen OA Auestad B Faerden A Hauge LJ Joa I Is there an optimal factor structure of the Positive and Negative Syndrome Scale in patients with first-episode psychosis? Scand J Psychol (2012) 54:160–5. 10.1111/sjop.12017 23252448

[35] Wallwork RS Fortgang R Hashimoto R Weinberger DR Dickinson D. Searching for a consensus five-factor model of the Positive and Negative Syndrome Scale for schizophrenia. Schizophr Res (2012) 137:246–50. 10.1016/j.schres.2012.01.031 22356801 PMC3351536

[36] Cannon-Spoor HE Potkin SG Wyatt RJ. Measurement of premorbid adjustment in chronic schizophrenia. Schizophr Bull (1982) 8:470–84. 10.1093/schbul/8.3.470 7134891

[37] van Mastrigt S Addington J. Assessment of premorbid function in first-episode schizophrenia: modifications to the Premorbid Adjustment Scale. J Psychiatry Neurosci (2002) 27:92–101. 11944510 PMC161638

[38] Bailer J Bräuer W Rey ER. Premorbid adjustment as predictor of outcome in schizophrenia: results of a prospective study. Acta Psychiatr Scand (1996) 93:368–77. 10.1111/j.1600-0447.1996.tb10662.x 8792907

[39] Buchanan RW Kirkpatrick B Clinical correlates of the deficit syndrome of schizophrenia. Am J Psychiatry (1990) 147:290–4. 10.1176/ajp.147.3.290 2309943

[40] Jaeger J Berns SM Czobor P. The multidimensional scale of independent functioning: a new instrument for measuring functional disability in psychiatric populations. Schizophr Bull (2003) 29:153–67. 10.1093/oxfordjournals.schbul.a006987 12908671

[41] Benjamini Y Hochberg Y Controlling the false discovery rate: a practical and powerful approach to multiple testing. J R Stat Soc B (1995) 57:289–300.

[42] Waters F Collerton D Ffytche DH Jardri R Pins D Dudley R Visual hallucinations in the psychosis spectrum and comparative information from neurodegenerative disorders and eye disease. Schizophr Bull (2014) 40:S233–45. 10.1093/schbul/sbu036 24936084 PMC4141306

[43] Skottun BC Skoyles J Dyslexia, direction selectivity and magnocellular sensitivity. Vision Res (2007) 47:1974–5. 10.1016/j.visres.2006.10.027 17316741

[44] Bauser DS Thoma P Aizenberg V Brüne M Juckel G Daum I. Face and body perception in schizophrenia: A configural processing deficit? Psychiatry Res (2012) 195:9–17. 10.1016/j.psychres.2011.07.017 21803427

[45] Chen Y. Abnormal visual motion processing in schizophrenia: a review of research progress. Schizophr Bull (2011) 37:709–15. 10.1093/schbul/sbr020 21436317 PMC3122297

[46] O’Donnell BF Swearer JM Smith LT Nestor PG Shenton ME McCarley RW. Selective deficits in visual perception and recognition in schizophrenia. Am J 1Psychiatry (1996) 153:687–92. 10.1176/ajp.153.5.687 8615416

[47] Kim J Park S Blake R. Perception of biological motion in schizophrenia and healthy individuals: a behavioral and fMRI study. PLoS One (2011) 6:e19971–19915. 10.1371/journal.pone.0019971 21625492 PMC3098848

[48] Marwick K Hall J. Social cognition in schizophrenia: a review of face processing. Br Med Bull (2008) 88:43–58. 10.1093/bmb/ldn035 18812413

[49] Young BG A phenomenological comparison of LSD and schizophrenic states. Br J Psychiatry (1974) 124:64–74. 10.1192/bjp.124.1.64 4822416

[50] Chapman J The early symptoms of schizophrenia. Br J Psychiatry (1966) 112:225–51. 10.1192/bjp.112.484.225 4957283

[51] Mathalon DH Ford JM. Neurobiology of schizophrenia: search for the elusive correlation with symptoms. Front Hum Neurosci (2012) 6:136. 10.3389/fnhum.2012.00136 22654745 PMC3360476

[52] Pinto L Goard MJ Estandian D Xu M Kwan AC Lee SH Fast modulation of visual perception by basal forebrain cholinergic neurons. Nat Neurosci (2013) 16(12):1857–63. 10.1038/nn.3552 24162654 PMC4201942

[53] Carnahan RM Lund BC Perry PJ Pollock BG Culp KR The anticholinergic drug scale as a measure of drug-related anticholinergic burden: associations with serum anticholinergic activity. J Clin Pharmacol (2013) 46(12):1481–6. 10.1001/archinte.163.22.2716 17101747

