# Supplementary material for: Self-Reported Visual Perceptual Abnormalities Are Strongly Associated with Core Clinical Features in Psychotic Disorders
Source: Front Psychiatry. 2018 Mar 12;9:69. doi: 10.3389/fpsyt.2018.00069 (PMC5858532; doi:10.3389/fpsyt.2018.00069)
Supplement: Supplementary file 1 [file data_sheet_1.docx]

**Supplementary Material**

**Table S1.** Bonn Scale for the Assessment of Basic Symptoms

| Bonn Scale Item Number | Description | Aimed Questions |
| --- | --- | --- |
| C.2.1.1 | Blurred/unclear vision | “Is your vision sometimes, either shortly or for a longer time, blurred and turbid, unclear or imprecise?” |
| C.2.1.2 | Transitory blindness- temporary blindness affecting whole visual field | “Have you ever experienced being blind for a while?” |
| C.2.1.3 | Partial seeing- only parts of object are perceived | “Can you sometimes see only parts of an object?” |
| C.2.2.1 | Hypersensitivity to light or certain optic stimuli | “Have you become sensitive to light?” |
| C.2.2.2 | Photopsias- seeing flashes or stars | “Do you sometimes see flashes of light or other very bright figures like sparks, stars, dots or flames?” |
| C.2.3.1 | Porropsia- Objects seem to be closer or farther away but unchanged in their size | “Do object seem to be farther away, to come closer or to move?” |
| C.2.3.2 | Micropsia- objects appear smaller than they are; Macropsia- objects appear bigger than they are | Did you ever feel like everything was very small and far away? |
| C.2.3.3 | Metamorphopsia – also called dysmorphopsia, when straight lines or objects appear wavy or deformed | “Do you sometimes see things peculiarly different, distorted, or deformed?” |
| C.2.3.4 | Metachromopsia- changes in color vision | Do you sometimes see things changed in color? |
| C.2.3.5 | Changes in the perception of the face/body of others | Do the faces or bodies of others appear different, changed? |
| C.2.3.6 | Changes in the perception of own face or body (so-called mirror phenomenon) | Does your own face or body sometimes appear different, changed? |
| C.2.3.7 | Pseudomovement of objects | Do objects or scenes ever seem to be moving? |
| C.2.3.8 | Double vision- double, oblique, slanting (sloping), and reversed vision | Do you sometimes see things two- or threefold, lopsided or crooked?” |
| C.2.3.9 | Disturbances of the estimation of distances | “Do things ever seem to be farther away, or closer to you than they really are?” |
| C.2.3.10 | Disintegration of the linearity of (objective) contours of objects- objects appear to be bending or curving | “Do straight outlines sometimes appear broken, curved or meandering?” |
| C.2.3.11 | Dysmegalopsia- inability to judge object sizes accurately | Do objects seem to be bigger on one side and smaller on the other than they really are? |
| C.2.3.12 | Persistence of stimuli - abnormally long-lasting optic stimuli and subsequent vision of things seen in reality minutes, hours or days before respectively | Do you sometimes feel like you’re seeing something for a long time? |

*Note.* The 17 vision items deriving from the Bonn Scale.
